# Comparative effects of CPAP and mandibular advancement devices in obstructive sleep apnea: an overview of systematic reviews

**DOI:** 10.1007/s11325-026-03784-y

**Published:** 2026-08-21

**Authors:** Marcela Carla Pereira do Nascimento, Ana Laura Vilela de Carvalho, Jessica Fernanda de Oliveira Lima Batista, Sérgio Soares da Silva, Belmiro Cavalcanti do Egito Vasconcelos, Sandra Lúcia Dantas Moraes, Eduardo Piza Pellizzer, Mônica Vilela Heimer

**Affiliations:** 1 Department of Pediatric Dentistry, Faculty of Dentistry, University of Pernambuco, 80 Norte Miguel Arraes de Alencar Avenue, Santo Amaro, Recife, Pernambuco 52071-035 Brazil; 2 Recife, Pernambuco Brazil; 3 Department of Oral and Maxillofacial Surgery, Faculty of Dentistry, University of Pernambuco, Recife, Pernambuco Brazil; 4 Division of Oral Rehabilitation, Faculty of Dentistry, University of Pernambuco, Recife, Pernambuco Brazil; 5 Department of Dental Materials and Prosthodontics, São Paulo State University, Araçatuba, São Paulo, Brazil

**Keywords:** Obstructive sleep apnea, Continuous positive airway pressure, Mandibular advancement device, Sleep apnea syndromes

## Abstract

**Purpose:**

This overview of systematic reviews aimed to compare continuous positive airway pressure and mandibular advancement devices for the treatment of obstructive sleep apnea by distinguishing objective physiological outcomes from patient-centered outcomes and critically appraising the methodological quality of the available evidence.

**Methods:**

Following PRISMA guidelines and PROSPERO registration (CRD42021268587), we systematically searched five databases to identify systematic reviews comparing continuous positive airway pressure and mandibular advancement devices in obstructive sleep apnea based on a PICO framework. The outcomes of interest included the Apnea–Hypopnea Index, oxygen desaturation index, treatment adherence, and quality of life. Methodological quality was assessed using AMSTAR-2, and overlap among reviews was evaluated using a citation matrix and the corrected covered area.

**Results:**

Fourteen systematic reviews were included. CPAP demonstrated greater efficacy than mandibular advancement devices in improving objective physiological outcomes, particularly the apnea–hypopnea index and oxygen desaturation indices. Both therapies showed comparable effects on daytime sleepiness, while some reviews reported better treatment adherence and more favorable patient-centered outcomes with MADs. Methodological quality was limited: no review was classified as high quality, and most were assessed as low or critically low quality. Analysis of the corrected coverage area indicated a high overlap between the primary studies included in the different reviews.

**Conclusion:**

Continuous positive airway pressure is more effective than mandibular advancement devices in improving the apnea–hypopnea index and oxygen desaturation index. However, mandibular advancement devices were associated with positive effects on quality of life and sleep, though certainty regarding the sustainability of these outcomes and their long-term efficacy remains limited in the current literature. Treatment choice should be individualized, and evidence should be interpreted with caution due to methodological limitations and the high overlap between the included systematic reviews.

**PROSPERO registration number:**

CRD2021268587.

**Supplementary information:**

The online version contains supplementary material available at https://doi.org/10.1007/s11325-026-03784-y.

## Introduction

Obstructive sleep apnea (OSA) is the most common form of sleep-disordered breathing and the second most common sleep disorder overall [1]. It is characterized by recurrent episodes of partial or complete upper airway obstruction, resulting in intermittent hypoxemia and sleep fragmentation [2]. Patients with OSA experience oxygen desaturation, reduced intrathoracic pressure, central nervous system excitation, and an increased risk of cardiovascular morbidity [2–4]. Untreated OSA has also been associated with metabolic dysfunction, cognitive impairment, daytime sleepiness, reduced quality of life, and an increased risk of traffic accidents [5, 6].

In adults, OSA is highly prevalent, although estimates vary according to diagnostic criteria, age, sex, and body mass index. Using a diagnostic criterion of an apnea–hypopnea index (AHI) of five or more events per hour and the 2012 AASM criteria, it has been estimated that 936 million individuals aged 30–69 years worldwide had OSA [7].

The most common clinical symptoms of OSA include snoring, fatigue, morning headaches, and gastroesophageal reflux [2, 8]. Beyond its clinical presentation, the primary modifiable risk factors are obesity, particularly when adiposity is concentrated around the neck and upper body, followed by smoking and alcohol consumption. Medical conditions such as hypothyroidism, polycystic ovary syndrome, and acromegaly have also been strongly linked to the development of OSA [9].

Therapeutic modalities for OSA include surgical interventions and non-invasive conservative measures [8], such as lifestyle modifications, continuous positive airway pressure (CPAP), and mandibular advancement devices (MAD) [1, 8, 9]. CPAP is considered the gold standard treatment for OSA. It delivers pressurized air, which helps to keep the airway open [8].

However, discomfort associated with CPAP use, as well as intolerance to the air pressure, can hinder patient adherence, compromising the effectiveness of the treatment [10]. As a result, the MAD, although primarily recommended for patients with mild-to-moderate OSA who are unable to adhere to or tolerate CPAP [9, 11] It has also been indicated for patients with severe OSA [10, 12], due to its higher reported adherence rates [10, 12].

This device works by advancing the jaw, which in turn moves the soft palate, tongue, and hyoid bone forward, enhancing the tonicity of the muscles that keep the airway open. This leads to a reduction in the AHI, an increase in oxygen saturation, and improvements in the primary symptoms associated with OSA [9, 13]. Although CPAP is generally more effective in reducing AHI and improving oxygenation, MAD may be better accepted and more consistently used by some patients [14].

While some systematic reviews have demonstrated the effectiveness of CPAP in treating OSA [8, 15]. Others have highlighted the efficacy of MAD [9, 13]. Furthermore, some systematic reviews found no significant differences between the two treatments [11, 16, 17].

These reviews differ in their search strategies, eligibility criteria, included studies, assessed outcomes, and methodological quality. Consequently, an overview of systematic reviews may help clarify not only the direction of the evidence but also the confidence that can be placed in the available findings by critically appraising the methodological quality of the existing reviews. Accordingly, the objective of this overview was to compare CPAP and MAD for the treatment of OSA through a comprehensive synthesis and critical appraisal of published systematic reviews.

## Materials and methods

### Search strategy

PubMed, Scopus, Embase, Web of Science, and the Cochrane Library databases were searched for studies published until July 2025 and updated in July 2026. To ensure the retrieval of relevant literature, specific filters for systematic reviews were applied across the databases during the search process. The reference lists of the selected articles from the databases and organizational websites were also examined. Two independent researchers (A. L.V. C. and S. S. S.) searched. Any discrepancies were resolved through discussion with a third author (M. V. H.) until a consensus was reached.

The search strategy combined keywords and MeSH descriptors “obstructive sleep apnea”, “continuous positive airway pressure”, and “mandibular advancement device” using the Boolean operators AND and OR, with adaptations for each database.

The search strategies were refined and standardized using the controlled vocabulary specific to each database (such as MeSH terms for PubMed and Emtree for Embase). To ensure clarity, the full electronic search strategy for PubMed is presented below, while the complete search strategies for the other databases are available in Supplementary Material 2. To ensure a comprehensive search, terms were adjusted as follows: ((apnea OR obstructive sleep apnea OR sleep apnea syndrome OR apnea syndrome OR snoring) AND (oral appliances OR mandibular advancement devices OR mandibular advancement appliances OR mandibular advancement splint OR mandibular advancement)) AND (cpap OR continuous positive airway pressure).

### Protocol and registration

This overview of systematic reviews followed the guidelines outlined in the Cochrane Handbook for Overviews of Reviews [18]. In addition, we adhered to the Preferred Reporting Items for Systematic Reviews and Meta-Analyses (PRISMA) framework and the PRISMA-Overviews extension, specifically developed for synthesizing multiple systematic reviews [19, 20]. The overview is registered in the Prospective Register of Systematic Reviews (PROSPERO—CRD42021268587).

### Eligibility criteria

The following PICO (problem/patient/population, intervention/indicator, comparison, outcome) question was formulated for this study: “Is there a difference in treatment efficacy between CPAP and the MAD for OSA?” The population studied consisted of patients with OSA. The intervention assessed was the treatment of OSA with CPAP, the comparison involved patients treated with the MAD, and the outcome measures were objective physiological parameters (AHI and ODI) and patient-centered outcomes, including treatment adherence, daytime sleepiness, and quality of life. No restrictions were imposed on language or year of publication.

### Inclusion criteria

Systematic reviews were selected based on the PICO question. The sole inclusion criterion was that the systematic reviews compared the use of CPAP with MAD for the treatment of OSA. Patients across all OSA severity levels and all MAD designs were included because this reflected the analytical approach adopted by the majority of the included systematic reviews.

### Exclusion criteria

The exclusion criteria were as follows: (1) Reviews that primarily included patients with central sleep apnea without clearly distinguishing the results for obstructive sleep apnea; (2) Reviews that did not provide separate analyses for the effects of CPAP and MAD; and (3) Reviews that did not report the relevant clinical outcomes of interest.

### Study selection

All articles were imported into the Rayyan Reference Manager for deduplication and analysis. Two reviewers (A. L. V. C. and S. S. S.) independently screened the titles and abstracts. Full-text articles were then assessed, and those that met the inclusion criteria were selected for final inclusion. Both reviewers performed this process. Any discrepancies were resolved through consultation with a third reviewer (M. V. H.). Inter-examiner agreement was evaluated using the kappa test to assess consistency between reviewers during the search and selection process.

### Data collection

One author (A. L. V. C.) collected the relevant information from the articles, and a second author (S. S. S.) independently reviewed all the collected data. The qualitative data collected included: author/year, registration/guide, quality assessment, included articles, study design, database, results (AHI, oxygen saturation, treatment adherence, and sleep quality), AMSTAR-2 rating, and study type.

### Quality assessment

The methodological quality of the systematic reviews was evaluated using the AMSTAR-2 tool, which consists of 16 items that assess key aspects of a review’s methodology, such as clarity of the research question, thoroughness of the search strategy, evaluation of risk of bias, and the appropriateness of statistical methods. AMSTAR-2 employs a domain-based rating system with seven critical and nine non-critical domains. Rather than providing a single overall score, it assigns weighted ratings based on these domains. Reviews are classified as “high quality” if they have no critical flaws, “moderate quality” if they have some weaknesses but no critical flaws, “low quality” if they contain a critical flaw, and “critically low quality” if they have multiple critical flaws, indicating that they should not be considered reliable or comprehensive [21].

### Overlap assessment

To assess overlap among the included systematic reviews, a citation matrix was constructed. Primary studies included in each systematic review were extracted and cross-tabulated against the reviews included in this overview. When duplicate citations or spelling variations referred to the same primary study, they were merged. When multiple reports referred to different studies, they were kept as separate primary studies.

The corrected covered area (CCA) was calculated to quantify the degree of overlap among the included systematic reviews using the following formula: $CCA=\frac{N-r}{rc-r}$, where N denotes the total number of primary-study occurrences across all reviews, r represents the number of unique primary studies, and c indicates the total number of systematic reviews. The degree of overlap was subsequently categorized as slight, moderate, high, or very high according to established thresholds [22].

## Results

**Fig. 1:**
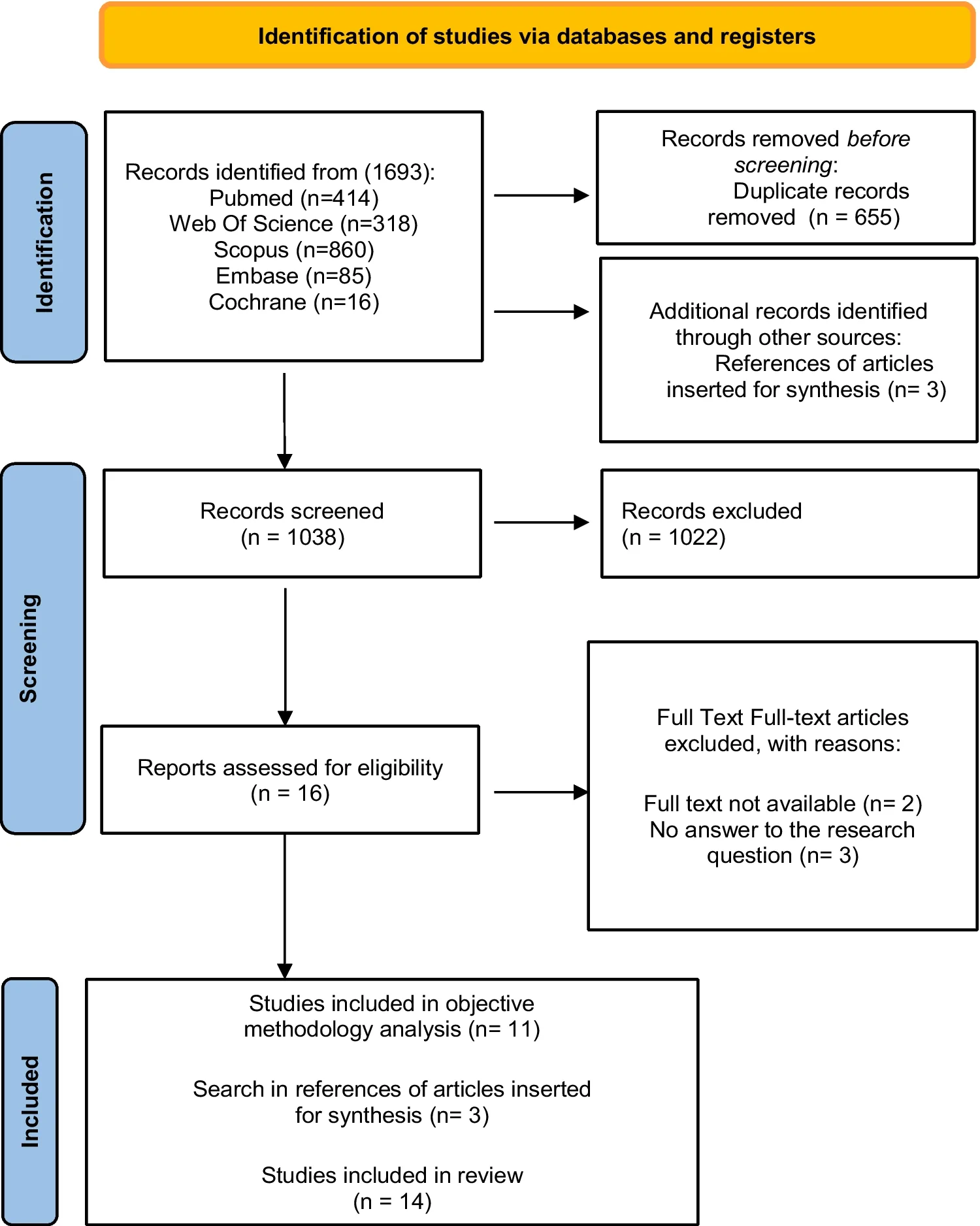
Prisma Flowchart

A total of 1693 articles were retrieved from the following databases: Cochrane, Embase, PubMed, Scopus, and Web of Science. After removing duplicates (*n* = 655), 1038 titles and abstracts were screened. Following this initial screening, 1022 articles were excluded, leaving 16 articles for full-text review. Of these, 11 articles met the inclusion criteria. Electronic searches were supplemented by hand-searching the reference lists of the selected articles. Three additional articles were identified through this process, resulting in a final sample of 14 systematic reviews (Fig. 1).

An analysis of inter-examiner consistency (between A. L. V. C. and S. S. S.) was conducted for each database, yielding the following kappa scores: PubMed (0.95), Cochrane Library (1.0), Scopus (0.86), Web of Science (0.86), and Embase (1.0), indicating a high degree of agreement between reviewers. Any discrepancies were resolved through consultation with a third reviewer (M. V. H.). A detailed description of each included study is provided in Table 1.

**Table 1:** Characteristics of the included studies

Author/Year	Meta-analysis model	Registration/Guide	Quality Assessment	Included Articles	Study Design	Database	Results	AMSTAR-2	Study Type
Lim et al. (2006)	Random-effects meta-analysis	No/NR	Jadad Score	17	Randomized clinical trials	PubMed/Medline -Cochrane	CPAP significantly reduced AHI compared with MAD in parallel studies (CI: 5.57 to 10.69) and crossover studies (CI: 6.38 to 9.56). CPAP also reduced oxygen desaturation index in parallel studies (CI: 2.55 to 6.64) and crossover studies (CI: 3.25 to 7.06). Daytime sleepiness was similar in parallel studies (*p* = 0.64), but favored CPAP in crossover studies (*p* < 0.001). Quality of life did not differ significantly (CI: −0.35 to 0.28)	Moderate	MA
Li et al. (2013)^15^	Random-effects meta-analysis	No/NR	Cochrane Handbook	14	Randomized and prospective clinical trials	-PubMed/Medline -Embase -Cochrane Library	Compared with MAD, CPAP significantly decreased AHI (*p* < 0.001) and oxygen desaturation index (*p* < 0.001) There was no significant difference between the two groups in excessive daytime sleepiness (*p* = 0.31 crossover trials/*p* = 0.09 parallel groups) and Treatment adherence (*p* = 0.26 crossover trials/*p* = 0.14 parallel groups)	Critically low	MA
Okuno et al. (2014)	Random-effects meta-analysis	No/NR	GRADE	5	Randomized and prospective clinical trials	-Pubmed/Medline -Cochrane Library -Japan Medical Abstracts Society	Compared with MAD, CPAP significantly decreased AHI (*p* < 0.0001) and oxygen desaturation index (*p* < 0.03) There was no statistically significant difference between CPAP and MAD in excessive daytime sleepiness (*p* = 0,09)	Low	MA
Iftikhar et al. (2016)	Network meta-analysis	Y/PRISMA	Cochrane Handbook	80	Crossover trials	-PubMed/Medline -Scopus -Web of Science -Cochrane Library	CPAP improved AHI compared with MAD (IC: −14,21 to −5,91) CPAP improved the oxygen desaturation index compared with MAD (IC: −13,04 to −2,59) There was no statistically significant difference between CPAP and MAD in excessive daytime sleepiness (IC: 0,64 to 1,18)	Low	NMA
Sharples et al. (2016)^6^	Pairwise meta-analysis	No/NR	Jadad Score	71	Crossover trials	-Pubmed/Medline -Embase -Science Citation Index	CPAP improved AHI compared with MAD (*p* < 0.001) MAD improved AHI compared with conservative management (*p* < 0.001) Both treatments improved excessive daytime sleepiness (MAD *p* < 0.001; CPAP *p* = 0.012)	Critically low	MA
Cammarotto et al. (2017)^18^	Random-effects meta-analysis	No/PRISMA	QUADAS-2	6	Crossover trials	-PubMed/Medline -Cochrane Library	CPAP improved AHI compared with MAD (*p* < 0.00001) CPAP improved the oxygen desaturation index compared with MAD (*p* < 0.00001) MAD improved daytime sleepiness levels compared to CPAP (*p* = 0.14). A low degree of heterogeneity was recorded	Critically low	MA
Liu et al. (2017)	Network meta-analysis	No/NR	Jadad Score	84	Crossover trials	-PubMed/Medline -Cochrane Library -Embase	CPAP improved AHI compared with MAD (IC: −16,05 to −1,50) CPAP improved the oxygen desaturation index compared with MAD (IC: 2,85 to 6,97) CPAP improved blood pressure compared with MAD (SBP IC: −11,34 to −7,81; DBP IC: −8,06 to −6,15; dSBP IC: −13,28 to −2,46)	Moderate	NMA
Schwartz et al. (2018)^1^	Random-effects meta-analysis	No/PRISMA	Cochrane Handbook	12	Crossover trials	-PubMed/Medline -Web of Science -Cochrane Library	CPAP significantly reduced the AHI (*p* < 0.05) compared to MAD There was no statistically significant difference between CPAP and MAD in excessive daytime sleepiness (*p* = 0.203)	Critically low	MA
Zhang et al*.* (2018)^10^		No/NR	Cochrane Handbook	16	Crossover trials	-PubMed/Medline -Web of Knowledge -Ovid -Dentistry & Oral Science Source -Cochrane Library -Embase	CPAP significantly reduced the AHI (*p* < 0.05) compared to MAD No significant difference between CPAP and MAD was found in excessive daytime sleepiness (*p* = 0.81)	Critically low	MA
Gao et al. (2019)	Network meta-analysis	No/NR	Cochrane Handbook	89	Randomized clinical trials	-PubMed/Medline -Embase -Cochrane Library -Google Scholar	Compared with the MAD, CPAP significantly reduced the AHI The exercise was ranked as the most effective in reducing excessive daytime sleepiness, followed by MAD and CPAP	Critically low	NMA
Li et al. (2020)^5^	Random-effects meta-analysis	No/NR	Jadad Score	14	Crossover trials	-PubMed/Medline -Embase -Cochrane Library	CPAP significantly decreased AHI compared with MAD (*p* < 0.001) There was no significant difference in excessive daytime sleepiness after therapy between the CPAP and MAD (*p* > 0.99)	Critically low	MA
Trzepizur et al. (2021)		No/NR	NR	4	Controlled trials	-PubMed/Medline -Embase -Cochrane Library	CPAP significantly decreased AHI compared with MAD (*p* < 0.0001) CPAP improved oxygen desaturation index compared with MAD (*p* < 0.03) There was no significant difference in excessive daytime sleepiness after therapy between the CPAP and MAD (*p* = 0.53) Treatment adherence was higher in the MAD group when compared to the CPAP group (*p* < 0.0001)	Critically low	MA
Pattipati et al. (2022)	Random-effects meta-analysis	No/PRISMA	NR	8	Crossover trials	-PubMed/Medline -Cochrane Library	Compared with MAD, CPAP was associated with a decrease in AHI (*p* < 0.01) and oxygen desaturation index (*p *< 0.01) There was no statistically significant difference between CPAP and MAD in excessive daytime sleepiness (*p* = 0.34)	Low	MA
Beri et al. (2023)	Random-effects meta-analysis	Y/PRISMA	Cochrane Handbook	19	Crossover trials	-PubMed/Medline -Scopus -Cochrane Library	CPAP significantly improved AHI compared with a MAD (*p* < 0.00001)	Critically low	MA

The systematic reviews included in this overview were published between 2006 and 2023. Regarding their analytical design, 11 reviews performed traditional pairwise meta-analyses [3, 13, 16, 19, 20, 23–28], while three used network meta-analysis (NMA) methodology [21, 29, 30] to compare treatments. The number of primary studies included in each review ranged from 4 [19] to 89 [29], with most consisting of randomized and non-randomized clinical trials. Two systematic reviews [21, 23] were registered in PROSPERO, and five [3, 21, 23–25] followed the PRISMA checklist for structuring their review. The remaining reviews [13, 16, 19, 20, 23, 26–28, 30] did not report adherence to any reporting guideline.

The criteria for assessing the risk of bias varied among the systematic reviews. Six reviews followed the Cochrane Handbook for analysis [3, 20, 21, 23, 27, 29]. Four reviews used the Jadad scale to assess risk of bias [13, 16, 31, 32]. One study utilized the GRADE [28] and the QUADAS-2 [24] scales, while two reviews assessed the risk of bias without employing any specific evaluation scale [19, 25].

Regarding the number of databases searched, three studies conducted their searches using only two databases (PubMed/MEDLINE and Cochrane Library) [33–35]. All other studies [3, 13, 16, 19–21, 23, 27–30] searched at least three databases (Table 1).

The methodological quality of the included reviews was evaluated using the AMSTAR-2 tool (Table 2). According to AMSTAR-2 ratings, two systematic reviews were classified as moderate quality [24, 35], three were rated as low quality [15, 23, 34], and nine reviews were classified as critically low quality [2, 9, 10, 14, 29, 33, 36, 37].

**Table 2:** AMSTAR-2 Scale Results

Item Study	1	2*	3	4*	5	6	7*	8	9*	10	11*	12	13*	14	15*	16	Rating Overall
Lim et al. 2006	Y	Y	N	Y	Y	Y	Y	Y	Y	N	Y	Y	Y	Y	Y	Y	Moderate
Li et al. (2013)	N	N	N	N	Y	Y	N	PY	N	N	Y	N	N	Y	Y	Y	Critically low
Okuno et al*.* (2014)	Y	PY	Y	Y	Y	Y	PY	Y	Y	N	Y	Y	Y	N	N	Y	Low
Iftikhar et al. (2016)	Y	Y	Y	PY	N	N	N	Y	Y	N	Y	Y	Y	Y	Y	Y	Low
Sharples et al*.* (2016)	Y	N	N	N	Y	Y	N	N	N	N	Y	N	N	Y	Y	Y	Critically low
Cammarotto et al. (2017)	Y	N	N	N	Y	N	N	PY	N	N	Y	N	N	Y	Y	Y	Critically low
Liu et al. (2017)	Y	PY	Y	Y	N	N	PY	Y	Y	N	Y	Y	Y	Y	Y	Y	Moderate
Schwartz et al. (2018)	N	N	N	N	Y	Y	N	PY	N	N	Y	N	N	Y	Y	Y	Critically low
Zhang et al. (2018)	N	N	N	N	Y	Y	N	N	N	N	Y	N	N	Y	Y	Y	Critically low
Gao et al. 2019	Y	PY	N	PY	Y	Y	N	PY	N	N	Y	Y	Y	Y	Y	Y	Critically low
Li et al. (2020)	Y	PY	N	N	Y	Y	N	PY	N	N	Y	N	N	N	Y	Y	Critically low
Trzepizur et al. (2021)	Y	PY	Y	PY	N	N	Y	Y	N	N	Y	Y	N	Y	N	Y	Critically Low
Pattipati et al. (2022)	Y	PY	Y	PY	N	N	N	Y	Y	N	Y	Y	Y	Y	Y	Y	Low
Beri et al. (2023)	Y	Y	Y	PY	Y	Y	PY	PY	Y	N	Y	N	N	Y	N	Y	Critically Low

**1**: PICO Question; **2**: Review Methods; **3**: Study Selection; **4**: Search Strategy; **5**: Duplicate Study Selection; **6**: Duplicate Data Extraction; **7**: List of Excluded Studies; **8**: Included Studies (Adequate Details); **9**: Assess Risk of Bias; **10**: Report on the Sources of Funding; **11**: Methods for Statistical Analysis; **12**: Impact of Risk of Bias in Individual Studies; **13**: Risk of Bias in Individual Studies; **14**: Heterogeneity Explanation Satisfactory; **15**: Investigation of Publication Bias; **16**: Report of Conflict of Interest

^*^Y yes, *PY* partial yes, *N* no (critical domain)

The critical domains most frequently unmet across the analyzed literature were Item 7 (absence of a complete list of excluded studies with explicit justifications) [2, 9, 10, 15, 16, 29, 33, 34, 36], which was unmet in 9 reviews, and Item 9 (inadequate assessment of the risk of bias in primary studies), which was not met in eight reviews [2, 9, 10, 14, 16, 29, 33, 36]. Additionally, for the critical Item 2 (prior protocol registration), five reviews achieved a full satisfactory rating [2, 10, 16, 29, 33]. Among the non-critical domains, Item 10 (reporting on the sources of funding of primary studies) represented a universal methodological failure, with 100% of the included reviews failing to report this information [2, 9, 10, 14–16, 23, 24, 29, 33–37].

A citation matrix was also constructed, identifying 189 distinct primary studies across the 14 included systematic reviews. The total number of primary-study occurrences was 439. The CCA was 10.18%, indicating a moderate degree of overlap among the included reviews.

Regarding the anatomical profile, most reviews reported only general anthropometric data (BMI) or omitted the assessment entirely**,** except Okuno et al. (2014), who focused on the design of the mandibular advancement device. When addressing subgroup statistical analyses based on anatomy or clinical observations, most authors failed to stratify findings by patient characteristics. The few stratifications identified were based on clinical constraints, such as severity according to the AHI [35] and baseline characteristics [14]. Regarding the direct assessment of the four pillars of pathophysiological phenotyping, Okuno et al. (2014) and Gao et al. (2019) were the only ones to consider the theoretical importance of these endotypes, yet they did not perform an actual assessment.

Physiological efficacy (AHI and ODI): All systematic reviews included in this overview indicate that CPAP achieves a significantly greater reduction in the AHI compared to MAD [2, 9, 10, 14–16, 23, 24, 29, 33–37]. CPAP also resulted in more pronounced improvements in oxygen desaturation index (ODI) levels [15, 16, 24, 33, 34]. However, regarding clinical symptom improvement, MAD was found to be as effective as CPAP, including in some patients with severe OSA [37].

Symptom outcomes (Sleepiness): The findings also suggest that CPAP and MAD have a comparable effect on reducing daytime sleepiness [2, 9, 10, 14, 15, 29, 33, 34].

Patient-centered outcomes (Quality of life): Both treatments led to improvements in quality of life and cognitive function, with some reviews reporting more favorable results for MAD than CPAP [9, 14, 16, 23]. Regarding blood pressure, only one included systematic review assessed this outcome, with results indicating that CPAP is more effective than MAD in reducing blood pressure [24].

Treatment adherence: No statistically significant differences were found in treatment adherence between the two groups, although patients generally preferred the long-term use of MAD [14, 29, 36].

The most frequently reported adverse effects in patients treated with CPAP included nasal congestion, facial muscle pain, eye irritation, and airway dryness [2, 16]. In cases of moderate to severe OSA, a sensation of suffocation during CPAP use was also reported [29]. For MAD, the most common adverse effects were dental pain, temporomandibular joint discomfort, excessive salivation, and morning mouth discomfort [2, 16].

## Discussion

All analyzed studies indicate that CPAP significantly reduces AHI and ODI compared to MAD [2, 9, 10, 14, 15, 23, 24, 29, 33–37]. However, when comparing these interventions in terms of secondary clinical outcomes, such as daytime sleepiness [2, 9, 10, 14, 15, 23, 29, 35, 36], quality of life, and cognitive function [6, 14, 16, 23, 29], no statistically significant difference was found. While the pooled analyses suggest comparable results for some patient-centered outcomes, this hypothesis of therapeutic equivalence must be interpreted with caution. Patient-centered outcomes are often heterogeneous and susceptible to reporting bias, as the greater physical comfort and convenience of MAD may lead to an overestimation of perceived improvement, despite the greater efficacy of CPAP in improving objective respiratory parameters. Furthermore, the sustainability of these subjective benefits remains unconfirmed, given the scarcity of long-term comparative evidence. This distinction is clinically relevant because treatment success in OSA depends not only on physiological efficacy, but also on adherence, tolerance, symptom control, patient preference, and long-term use. Thus, MAD emerges as a conditional option for individuals intolerant to positive airway pressure or those with mild-to-moderate OSA [14, 16, 29]. However, these secondary outcomes do not diminish the consistently greater efficacy of CPAP in improving objective respiratory outcomes.

This apparent discrepancy highlights an important clinical consideration**:** while CPAP remains the reference standard for physiological normalization, its real-world effectiveness may be limited by patient adherence [2, 9, 10, 14–16, 23, 24, 29, 33–37]. Conversely, the higher comfort and overall acceptance of MAD may contribute to better treatment adherence, which may partially offset their lower efficacy in objective respiratory outcomes and contribute to improvements in patient-centered outcomes and quality of life [9, 14, 16, 23]. While some pooled analyses [14, 16, 29] suggest that MAD could be considered in severe cases when CPAP is not tolerated, this approach must be individualized due to its lower efficacy in improving objective respiratory outcomes**.**

The greater adherence to MAD may explain the comparable outcomes to CPAP regarding patients’ quality of life and sleep quality [10, 35]. Patients often prefer MAD over CPAP due to the discomfort and inconvenience associated with CPAP mask use and machine setup [16, 26, 27]. Additionally, the design of MADs allows for customization, which contributes to higher adherence rates [28, 30]. This flexibility enables adaptation of the therapeutic plan to each patient’s individual needs, considering anatomical factors and allowing for adjustments in the degree of mandibular advancement [30]. However, this variability in appliance design may have contributed to the heterogeneity observed across the included reviews. In addition, titration protocols, degree of mandibular advancement, and manufacturing characteristics were not consistently reported.

Although CPAP is considered the first-line treatment for OSA [2, 9, 10, 14–16, 23, 24, 29, 33–37], due to its function as a pneumatic stent that stabilizes the upper airway and prevents its collapse during sleep, its effectiveness is highly dependent on patient adherence. The low adherence rate associated with CPAP is influenced by factors such as limited access to polysomnography for proper pressure titration, airway mucosa dryness, social constraints, and insufficient follow-up to address potential issues during use [35]. In contrast, treatment discontinuation rates are significantly lower with MAD, primarily due to the adverse effects commonly associated with CPAP [31].

Interestingly, a higher AHI at initial diagnosis is associated with better CPAP adherence, as patients with more severe OSA are typically more symptomatic and more attentive to the disease’s consequences [15, 32]. However, for CPAP therapy to be effective, it must be used every night for more than six hours during sleep [33, 35]. If CPAP treatment does not achieve the desired therapeutic success, similar outcomes can be obtained with MAD [9, 14, 15, 29, 33].

The MAD is primarily indicated for mild and moderate OSA [15, 37]. However, MAD may also be considered for selected patients with severe OSA who are intolerant of CPAP [10, 14, 37], especially in cases involving mandibular retrognathia [37, 38]. Mandibular advancement devices reduce the need for positive airway pressure and may improve treatment acceptance and adherence [15, 31]. Patients often prefer MAD when given a choice and use it more consistently than CPAP [16, 29].

Regarding long-term efficacy, evidence derived from the included systematic reviews remains limited, as most pooled clinical trials monitor patients for only a few months. To contextualize this limitation, data from an individual prospective observational cohort study (Vecchierini et al., 2021) [37] suggested that while MAD clinical success can be sustained for up to five years, a gradual decline in efficacy occurs over time, varying according to initial disease severity. Similarly, long-term adherence remains a critical variable; individual observational data (Yu et al., 2023) [39] reported a subtle patient preference for MAD over CPAP regarding daily hours of use over extended periods. Nevertheless, these specific observations underscore the need for future systematic reviews to focus on long-term standardized data, as the current review-level literature cannot yet establish definitive longitudinal patterns.

In addition to effectiveness, patient adherence plays a crucial role in MAD therapy. Vecchierini et al. (2021) [38] also evaluated MAD usage compared to CPAP. Over several years, 91.3% of the 331 patients monitored reported using MAD for at least six hours per night. Furthermore, 96.5% of patients expressed a preference for MAD over CPAP, highlighting its acceptability and potential for sustained use. Similarly, Yu et al. (2023) [39], reinforced the preference for MAD over CPAP, with patients using MAD for an average of 1.1 h longer per night than CPAP. This increased adherence is particularly important, as therapeutic success with MAD is closely linked to consistent use. The study also suggested that the reduction in AHI achieved with MAD remains stable over time. However, variability across studies highlights the need for further research to standardize long-term outcomes and compare MAD’s effectiveness with other treatment modalities.

Regarding blood pressure regulation, the evidence derived from the included network meta-analysis indicated that CPAP was more effective than MAD [24]. This finding, however, contrasts with findings from a recent primary clinical trial (Ou et al., 2024) [40], which reported blood pressure reductions in a specific cohort of patients with moderate-to-severe OSA treated with MAD**.** This discrepancy may reflect methodological differences, as systematic reviews synthesize data from diverse populations with varying adherence levels, whereas individual trials evaluate more specific populations under controlled conditions**.** Ultimately, within the scope of this overview, the synthesized review-level evidence supports CPAP over MAD for blood pressure reduction, while the effect of MAD on blood pressure requires further validation through robust, high-quality meta-analyses.

A study by Beri et al. (2023) [37] compared the effects of CPAP, MAD, physical training, and weight loss on reducing AHI and daytime sleepiness. The results showed that CPAP significantly reduced AHI in patients. However, no statistically significant difference was found when comparing CPAP to physical training in terms of AHI reduction. Regarding daytime sleepiness, MAD yielded the most favorable outcomes. Similar improvements were observed with CPAP followed by weight loss. These findings highlight the importance of lifestyle modifications in patients diagnosed with OSA and suggest that physical activity should be actively promoted in clinical settings as an adjunctive treatment to CPAP or MAD, given its positive impact on the investigated outcomes [15, 37].

The PROSPERO registration tool, available since February 2011, allows for free searches of systematic reviews to maintain transparency. Despite its availability, only two of the 14 articles included in this overview were registered with PROSPERO [15, 37], even though all were published after its implementation. Registering a systematic review improves the quality of the data and minimizes the risk of bias [19]. However, to register with PROSPERO, authors must follow a protocol that requires justification of all methodological decisions [19], which may explain why many authors chose not to register their reviews. [2, 9, 10, 14, 16, 23, 24, 29, 33–35].

Additionally, adhering to the PRISMA guidelines improves the quality of systematic reviews. However, nine of the 14 included reviews did not use PRISMA as a guide [9, 10, 14, 16, 23, 24, 29, 35, 36]. Three of these reviews were conducted before the release of the PRISMA protocol [23, 29, 35] and six [9, 10, 14, 16, 24, 36]] did not follow PRISMA after its publication, suggesting potential flaws in the evaluation of included articles [19]**.**

A limitation of the studies included in this overview is the restricted database search. While well-established databases like PubMed and Cochrane Library were frequently searched [2, 9, 10, 14, 15, 23, 24, 29, 33–37], fewer studies searched other databases such as Embase [9, 10, 14, 16, 24, 29, 36]; Scopus [15, 37], and Web of Science [2, 15]. Limiting the search to a few databases may lead to gaps in the literature and less representative findings. Methodological guidelines, such as PRISMA and Cochrane, recommend extensive searches across multiple databases to ensure all relevant studies are considered [19, 41].

The methodological limitations revealed by the AMSTAR-2 assessment, specifically the absence of a dedicated list of excluded studies (Item 7) and the inadequate assessment of risk of bias (Item 9), may increase the risk of bias in the included reviews**.** Furthermore, the consistent lack of reporting of funding sources for primary studies (Item 10) limits the assessment of potential conflicts of interest. Consequently, the predominance of low and critically low methodological quality reduces confidence in the available evidence, and current review conclusions should be applied in clinical practice with caution.

The risk of bias was identified as a critical flaw in nine studies [2, 9, 10, 14, 16, 29, 33, 36] due to issues with randomization, allocation sequence, and study selection. As a result, the quality of these reviews was rated as critically low according to the AMSTAR-2 tool, exacerbated by heterogeneity in reported results and statistical variations.

From a methodological standpoint, the citation matrix revealed a moderate degree of overlap among the included reviews, indicating a relevant reliance on the same primary studies. Although even this moderate volume of published systematic reviews might suggest a diverse body of literature, the collective evidence largely stems from the same pool of primary articles.

A critical finding of this overview is the scarcity of evaluations guided by the four pathophysiological pillars/endotypes of obstructive sleep apnea. Despite the growing call for personalized sleep medicine, available meta-analyses still rely almost exclusively on clinical severity metrics, including the AHI, and general anthropometric data, including BMI, to compare the efficacy of CPAP and oral appliances. This approach highlights a significant methodological gap, as it limits the understanding of how different phenotypic profiles may influence therapeutic response.

Another limitation is that OSA severity and symptom burden were not uniformly standardized across the included reviews. While some reviews classified patients based on AHI, ODI, or ESS, others included patients with varying degrees of OSA severity. This lack of standardization may have contributed to heterogeneity and limits the direct comparability of CPAP and MAD outcomes across reviews.

Conducting an overview of systematic reviews is a valuable approach for evaluating and summarizing results in a single document that can guide health professionals and policymakers. This approach provides a comprehensive synthesis of the highest level of available evidence, facilitating evidence-informed clinical and policy decision-making. However, the limitations of this study include the lack of detailed analysis of primary studies, reliance on data from existing systematic reviews, and heterogeneity among selected studies, which increase the risk of bias. Future systematic reviews should follow established methodological standards, including protocol registration and adherence to the PRISMA reporting guidelines, to improve methodological quality and strengthen the certainty of the available evidence. Further research should also investigate the influence of anatomical and pathophysiological phenotypes, as well as long-term clinical outcomes, on the comparative effectiveness of CPAP and MAD.

## Conclusion

The evidence synthesized in this overview demonstrates that CPAP is clinically superior to MAD in reducing objective respiratory parameters, specifically the apnea–hypopnea index and oxygen desaturation index. However, both therapies showed comparable results for several patient-centered outcomes, although these findings should be interpreted with caution because of the methodological limitations of the available evidence. Furthermore, the AMSTAR-2 assessment revealed that none of the included systematic reviews exhibited high methodological quality, and the citation matrix demonstrated a moderate degree of overlap, indicating that many reviews included the same primary studies. Future high-quality primary studies and systematic reviews are needed to strengthen the evidence, particularly regarding long-term outcomes and patient phenotyping. Consequently, while CPAP remains the primary recommendation for respiratory normalization, MAD serves as a valuable alternative for patients with mild-to-moderate OSA or those who are intolerant of or unable to adhere to pressure therapy.

## Supplementary information

Below is the link to the electronic supplementary material.

Supplementary file1 (XLSX 27 KB)

Supplementary file2 (DOCX 9 KB)

## Data Availability

All data analyzed in this study are publicly available in the published literature. No new data were generated or analyzed.
