# Supplementary material for: Comparative effects of CPAP and mandibular advancement devices in obstructive sleep apnea: an overview of systematic reviews
Source: Sleep Breath. 2026 Aug 21;30(5):240. doi: 10.1007/s11325-026-03784-y (PMC13493485; doi:10.1007/s11325-026-03784-y)
Supplement: Supplementary file 1 [file 11325_2026_3784_MOESM2_ESM.docx]

| PUBMED – 1.103  filtro - 414 | ((apnea OR obstructive sleep apnea OR sleep apnea syndrome OR apnea syndrome OR snoring) AND (oral appliances OR mandibular advancement devices OR mandibular advancement appliances OR mandibular advancement splint OR mandibular advancement)) AND (cpap OR continuous positive airway pressure) |
| --- | --- |
| WEB OF SCIENCE – 1.139 | apnea OR obstructive sleep apnea OR sleep apnea syndrome OR apnea syndrome OR snoring (All Fields) and oral appliances OR mandibular advancement devices OR mandibular advancement appliances OR mandibular advancement splint OR mandibular advancement (All Fields) and cpap OR continuous positive airway pressure (All Fields) |
| SCOPUS – 3.189 | ( ALL ( apnea OR obstructive sleep apnea OR sleep apnea syndrome OR apnea syndrome OR snoring ) AND ALL ( oral appliances OR mandibular advancement devices OR mandibular advancement appliances OR mandibular advancement splint OR mandibular advancement ) AND ALL ( cpap OR continuous positive airway pressure ) ) |
| COCHRANE - 16 | apnea OR obstructive sleep apnea OR sleep apnea syndrome OR apnea syndrome OR snoring in All Text AND oral appliances OR mandibular advancement devices OR mandibular advancement appliances OR mandibular advancement splint OR mandibular advancement in All Text AND cpap OR continuous positive airway pressure in All Text - (Word variations have been searched) |
| EMBASE - | ('apnoea'/exp OR 'apnoea' OR 'periodic apnea'/exp OR 'periodic apnea' OR 'periodic apnoea'/exp OR 'periodic apnoea' OR 'apnea'/exp OR 'apnea') AND ('mandibular advancement' OR 'sleep apnea appliance') AND ('constant positive airway pressure' OR 'constant positive pressure breathing' OR 'constant positive pressure ventilation' OR 'continous positive airway pressure' OR 'continuous positive airway pressure ventilation' OR 'continuous positive pressure breathing' OR 'continuous positive pressure ventilation' OR 'cpap' OR 'cppb' OR 'cppv' OR 'continuous positive airway pressure') |
